# Does radiotherapy increase the risk of colorectal cancer among prostate cancer patients? A large population-based study

**DOI:** 10.7150/jca.44726

**Published:** 2020-08-25

**Authors:** Chung-Han Ho, Kuo-Chen Cheng, Chien-Ming Chao, Chih-Cheng Lai, Shyh-Ren Chiang, Chin-Ming Chen, Kuang-Ming Liao, Jhi-Joung Wang, Po-Huang Lee, Chao-Ming Hung, Chi-Ming Tai, Chong-Chi Chiu

**Affiliations:** 1 Department of Medical Research, Chi Mei Medical Center, Tainan 71004, Taiwan; 2 Department of Hospital and Health Care Administration, Chia Nan University of Pharmacy and Science, Tainan 71710, Taiwan; 3 Cancer Center, Wan Fang Hospital, Taipei Medical University, Taipei 11696, Taiwan; 4 Department of Internal Medicine, Chi Mei Medical Center, Tainan 71004, Taiwan; 5 Department of Safety, Health and Environment, Chung Hwa University of Medical Technology, Tainan 71703, Taiwan; 6 Department of Intensive Care Medicine, Chi Mei Medical Center, Liouying 73657, Taiwan; 7 Department of Intensive Care Medicine, Chi Mei Medical Center, Tainan 71004, Taiwan; 8 Department of Dental Laboratory Technology, Min-Hwei College of Health Care Management, Tainan 73657, Taiwan; 9 Department of Internal Medicine, Kaohsiung Veterans General Hospital, Tainan Branch, Tainan 71004, Taiwan; 10 Departments of General Education, Chia Nan University of Pharmacy and Science, Tainan 71710, Taiwan; 11 Department of Internal Medicine, Chi Mei Medical Center, Chiali 72263, Taiwan.; 12 AI Biomed Center, Southern Taiwan University of Science and Technology, Tainan 71005, Taiwan; 13 Department of Surgery, E-Da Hospital. I-Shou University, Kaohsiung 82400, Taiwan; 14 Department of General Surgery, E-Da Cancer Hospital, I-Shou University, Kaohsiung 82400, Taiwan; 15 Department of Internal Medicine, E-Da Hospital, I-Shou University, Kaohsiung 82400, Taiwan

**Keywords:** secondary colorectal cancer, prostate cancer, radiotherapy, radical prostatectomy, large population-based study

## Abstract

**Objective:** The survival of prostate cancer (PC) patients after radiotherapy (RT) has improved over time, but it raises the debate of increased risk of secondary colorectal cancer (SCRC). This study aimed to assess whether RT for PC treatment increases the risk of SCRC in comparison with radical prostatectomy (RP).

**Methods:** A population-based cohort of PC patients treated only with RT or only with RP between January 2007 and December 2015 was identified from the Taiwan Cancer Registry. The incidence rate of SCRC development was estimated using Cox regression model.

**Results:** In this study, total 8,797 PC patients treated with either RT (n = 3,219) or RP (n =5,578). Patients subjected to RT were elder (higher percentage of 70≧years, *p* < 0.0001) and more advanced clinically (stage III: 22.90% vs. 11.87%; stage IV: 22.15% vs. 13.80%, *p* < 0.0001), compared to those subjected to RP. More patients subjected to RT had a much higher percentage of autoimmune disease (22.34% vs. 18.75%, *p* < 0.0001) and osteoarthritis and allied disorders (16.31% vs. 12.98%,* p* < 0.0001). Besides, RT patients had a higher percentage of underlying Crohn's disease (0.25% vs. 0.05%, *p* = 0.0230). Although almost all selected factors were not statistically significant, they presented the positive risk of SCRC for those under RP compared with those among RT. Besides, for PC patients in clinical stage I and II, patients with RP may have borderline significantly protective effects of SCRC compared with those under RT (stage I, HR: 0.14; 95% C.I.:0.01-1.39; *p* = 0.0929; stage II, HR: 1.92; 95% C.I.:0.93-3.95; *p* = 0.0775). Kaplan-Meier curves for a 3-year-period, which demonstrated no statistical difference in the risk of SCRC free between PC patients undergoing RT and RP (*p* = 0.9766).

**Conclusion:** Whether or not pelvic RT for PC is associated with an increased risk for SCRC on a population-based level remains a matter of considerable debate. From a clinical perspective, these PC survivors should be counseled accordingly and received continued cancer surveillance with regular colonoscopy follow-up.

## Introduction

Prostate cancer (PC) is among the most common cancers in men worldwide. It is estimated at about 1,600,000 new cases and causes about 366,000 deaths every year 1. Due to the widespread adoption of the prostate-specific antigen test, the majority of PC are diagnosed at an early stage 2. Most of those non-metastatic cancers will be treated with radiotherapy (RT) or radical prostatectomy (RP) 3. However, radiation-induced secondary malignancies are a known but infrequent complication of RT, which would be expected to occur within the irradiated field 4. In clinical practice, RP and RT have similar oncologic outcomes 5. However, many controversies still exist regarding related risks after both therapeutic choices, which are associated with different quality-of-life detriments 6-15. RP predisposes to surgical mortality 16, 17, which may be avoided with RT. Conversely, RT may predispose to secondary malignancies in long-term follow-up 18-25. Thus, one must consider the side effects of each therapy before informed decisions can be made 26, uniquely identifying whether patients at increased risk of developing a secondary colorectal cancer (SCRC) would help to optimize treatment of the primary PC and to diagnose SCRCs promptly 2.

In the past, only scanty studies tried to investigate the risk of developing an SCRC 27-33. The literature regarding the risk of RT related SCRC is inconsistent. Some authors have demonstrated an increased risk of rectal cancer 20, 34, but others have not 23, 35. Similarly, this contradiction also existed in patients of colon cancer 3, 19, 20. However, many previous studies had short follow-up times and thus included many patients who were not yet at risk of radiation-induced neoplasia 3. Besides, most studies were originated from Western countries, and it is generally believed that the incidence of PC is lower in Asian countries 36.

Our study focused on comparing the incidence of SCRC after RT and RP for PC. The relative risk of developing an SCRC was analyzed. Besides, we compared the clinical characteristics and oncologic outcomes of PC patients after RP or RT treatment in a nationwide setting.

## Materials and Methods

### Study design and data sources

This study analyzed administrative claims data obtained from the Taiwan Bureau of National Health Insurance (BNHI). Because the BNHI was the sole payer in Taiwan, the BNHI data set covered the information of more than 23 million people, which was assumedly the most comprehensive and reliable data source for the study. Besides, the Taiwan Cancer Registry (TCR) was used to identify cancer patients. TCR established in 1979 to monitor the Taiwan's cancer incidence and the mortality rates. From the 20 items of all newly diagnosed cancers information (short-form database), TCR added detailed diagnosis and treatment items (long-form database) in 2002 for cancer types as oral cavity and pharynx (except nasopharynx), colon and rectum, liver, lung, breast, and cervix. In 2007, the prostate cancer was also added in the long-form database 37, 38.

The subjects of this study were selected from the TCR. The history of the diagnosis claims among those patients obtained from the national health insurance research database (NHIRD). The NHIRD contained a registry of contracted medical facilities, a registry of board-certified physicians, and monthly summaries for all inpatient claims. Since the analysis was limited to aggregate secondary data that could not be used to identify the patients, this study was exempt from full review by the internal review board at this institution. However, the study protocol conformed to the ethical standards established by the Declaration of Helsinki in 1964, which did not require written or verbal consent for data linkage studies.

### Study patients

The TCR included all cases of patients who were derived from the International Classification of Disease codes (ICD-9-CM) and those following a principal diagnosis of malignant neoplasm of the prostate (ICD-9-CM diagnosis code 185) as primary cancer (n = 36,543) from January 2007 to December 2015. It was staged according to the Tumor, Node, Metastasis (TNM) classification. After including either RP (ICD-9-CM procedure code 60.5) or RT (ICD-9-CM procedure code 92.29) as treatment modalities, these samples were eligible patients in this study. However, the analysis further excluded patients with no diagnosis of secondary malignant neoplasm of colon or rectum (ICD-9-CM diagnosis codes 153.0, 154.0, 154.1, 154.2, 154.3, 154.8, 230.3, 230.4, 230.5, 230.6 or 197.5), no correct clinical information, or patients receiving neither or both RT and RP.

**Figure 1:**
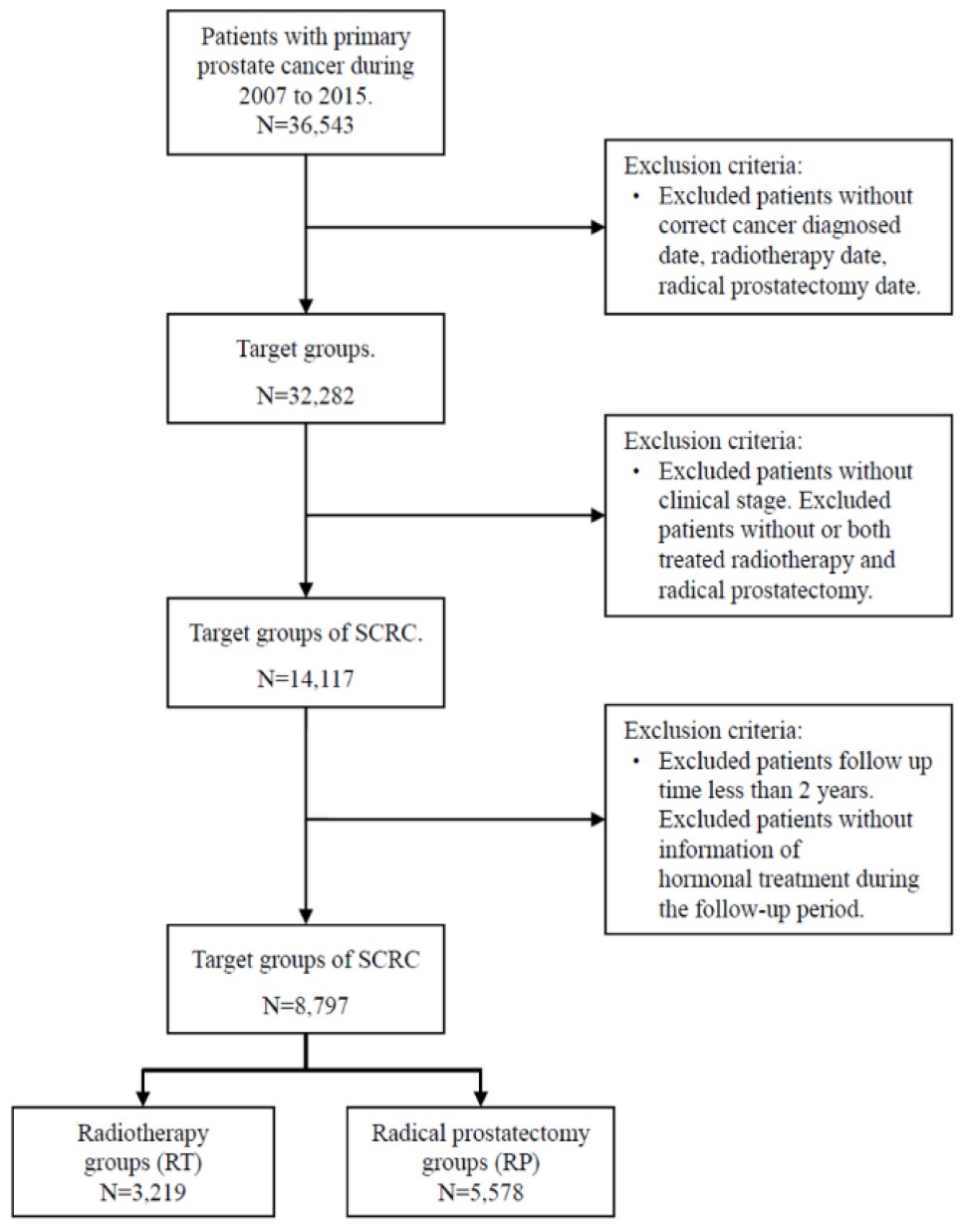
Flowchart for study subject selection

It was generally considered that secondary primary cancer required a latency period at least two months after the primary diagnosis to eliminate synchronous primary cancers 39. RT was not known to cause SCRC in the short term. Thus, we extended the analyses of the incident cases that occurred within 36 months after RP or RT delivery in our study design. Because comorbidities may result in premature mortality and may not allow the development of secondary malignancies, we adjusted for baseline comorbidities, including of Crohn's disease (ICD-9-CM: 569.81), diabetes mellitus (DM) (ICD-9-CM: 250), end-stage renal disease (ESRD) (ICD-9-CM: 585), hyperlipidemia (ICD-9-CM: 272), liver cirrhosis (LC) (ICD-9-CM: 571.5), chronic obstructive pulmonary disease (COPD) (ICD-9-CM: 490-496), and autoimmune diseases (ICD-9-CM: 274.9, 279.4, 710, 714, 715) which including gout (ICD-9-CM: 274.9), diffuse diseases of connective tissue (ICD-9-CM: 710), osteoarthritis and allied disorders (ICD-9-CM: 715), and rheumatoid arthritis (ICD-9-CM: 714). For avoiding the potential misclassification bias, the cases without correct diagnosis date, RT date, RP date, and information of the clinical stage were excluded. Cases who both received RT or RP during the study period were also excluded. Besides, the cases whose follow-up time fewer than two years were excluded to reduce the effect of a possible competing risk. The flowchart of study subjects' selection presented as Figure 1.

### Statistical Analysis

Our study population was divided into two subgroups according to the treatment modalities for PC, including RP and RT. For analyses, RP patients were considered controls. Conversely, patients treated with RT during the same period were considered cases.

Pearson's Chi-squared analysis was used to compare the age, clinical stages of PC, and comorbidities between patients with PC treated by RT and those treated by RP. Analyses were stratified by age at PC diagnosis (early-onset <60 years, 60-69 years or late-onset >70 years). Age was dichotomized into these groups because these cutoffs have commonly been used in demographic and epidemiologic studies, as well as statistics on the elderly. In addition, Wilcoxon's rank sum test was used to estimate the difference between PC patients treated by RT and those treated by RP for the variables, time to SCRC and time to death.

The trend of SCRC free incidence was calculated using the Kaplan-Meier method. Kaplan-Meier analyses addressed the time to the diagnosis of SCRC after having received either RP or RT. We calculated Kaplan-Meier curves representing the incidence rate of SCRC free development from PC diagnosis. The trend differences between RP and RT groups were compared with the log-rank test. In order to estimate the rare events and control the potential confounders, Cox proportional hazards model with Firth's penalized likelihood approach was constructed to adjust the age at diagnosis, PC clinical stages, hormone therapy, and comorbidities. The stratified analysis of each interested variable was also presented. In addition, we also estimated the other event, rectal cancer, a subtype of colorectal cancer. For all comparisons, a *p*-value of less than 0.05 was considered statistically significant. The statistical software, Statistical Analysis System (SAS) (version 9.4; SAS Institute, Inc, Cary, NC, USA), was used to perform all statistical analyses. Survival curves were generated using STATA version 12 (StataCorp LP, College Station, TX, USA).

## Results

Table 1 showed the descriptive characteristics of 8,797 PC patients treated with either RT (n = 3,219) or RP (n = 5,578) in this study. Patients subjected to RT were elder (higher percentage of ≥70years,* p* < 0.0001) and more advanced clinically (stage III: 22.90% vs. 11.87%; stage IV: 22.15% vs. 13.80%, *p* < 0.0001), compared to those subjected to RP. Besides, more patients subjected to RT had a much higher percentage of autoimmune diseases (22.34% vs. 18.75%, *p* < 0.0001) and osteoarthritis and allied disorders (16.31% vs. 12.98%, *p* < 0.0001). Besides, RT patients had a higher percentage of underlying Crohn's disease (0.25% vs. 0.05%, *p* =0.0230), despite the patient number was scarce (8 vs. 3).

Table 2 showed the results of the adjusted hazard ratio of SCRC after therapy by a different stratum of selected confounding factors. Although almost all selected factors were not statistically significant, they presented the definite risk of SCRC for those under RP compared with those among RT. Besides, for PC patients in clinical stage I and II, patients with RP may have borderline significantly protective effects of SCRC compared with those under RT (stage I, HR: 0.14; 95% C.I.:0.01-1.39; *p* = 0.0929; stage II, HR: 1.92; 95% C.I.:0.93-3.95; *p* = 0.0775). Similarly, no significant statistical difference was noted while only rectal cancer patient group was analyzed individually from all our SCRC patients.

**Figure 2:**
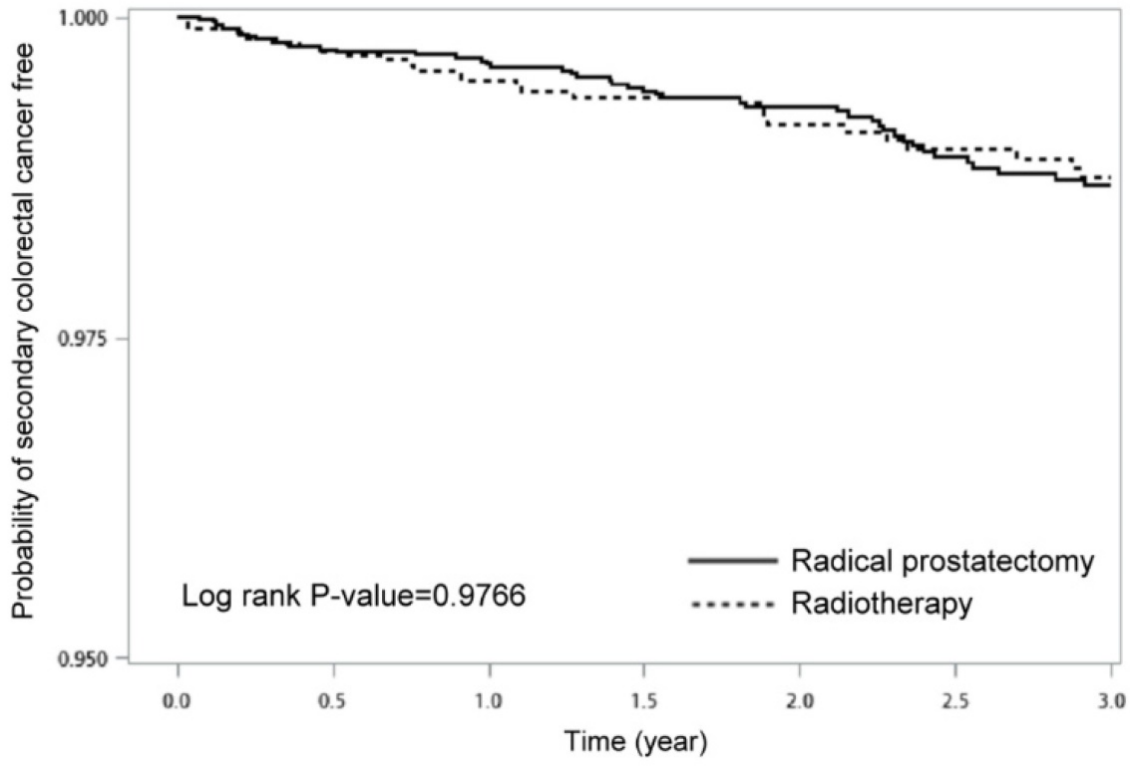
Probability of SCRC free between prostate cancer patients with radical prostatectomy and those with radiotherapy

Figure 2 displayed the Kaplan-Meier curves for a 3-year-period, which demonstrated no statistical difference in the incidence rate of SCRC free between PC patients undergoing RT and RP (*p* = 0.9766).

## Discussion

In the United States, 11% of men are diagnosed with PC over their lifetime, with the incidence generally rising with age; there are an estimated 165,000 cases and 29,000 deaths annually 40.

Several different treatment strategies could be considered for patients with PC, including active surveillance, RP, and radiation therapy (RT and/or brachytherapy). Strategy selection by physicians mainly depends on cancer risk assessment and possible residual lifespan as well as patient preferences 39. RP for invasive PC is associated with positive margin rates in 10% to 50% of resected specimens. Post-operative RT may benefit patients who have an organ-confined PC with positive margins 41. RT provides the patients with advanced cancer an effective treatment, and it is expected to inhibit the potential of cancer cell multiplication and lead to cell death 42. However, the long-life expectancy of these PC patients exposes them to the possibility of developing SCRC 39, which would be considered one of the most worrisome adverse effects of RT 26.

Among cancer of different organs, patients with PC provide an excellent opportunity to study the late effects of RT, because sufficiently large numbers of patients are available for study, the life expectancy of these men well exceeds 25 years 43, non-irradiated patients can be compared, and radiation doses to organs other than the prostate can be estimated accurately in prospective studies.

Factors associated with the development of rectal toxicity after RT for PC are variable and may be categorized as they relate to radiation delivery or patient characteristics 44. Although the correlation between radiation dose and rectal toxicity is somewhat intuitive and well accepted, the effect of radioresistance and repopulation at the primary site and/or at the malignant areas cause a paramount challenge in cancer control 42.

Secondary cancers were primarily defined as having different histologic features from primary cancer according to a criterion by Warren and Gated to distinguish recurrence or metastasis from the primary 45. Besides the possible effect of RT, factors contributing to the development of primary cancer probably also play a specific key in secondary cancer development 46. Establishing an association between malignancies may shed light on possible shared carcinogenic mechanisms, reveal an impact of treatment for one on the development of the other, and help develop evidence-based surveillance protocols 22. Some studies mentioned that cancer survivors are at increased risk of developing second cancer compared with an age- and the sex-matched general population 47. This increased risk is multifactorial and has been explained by several factors, such as lifestyle factors, genetic susceptibility, and administered chemotherapy or RT. For example, men with PC had a higher risk of developing bladder, kidney, soft tissue, and endocrine cancers. However, some studies showed converse results. Some researchers observed that PC survivors had an overall 40% lower risk of secondary primary cancer development in comparison with the general male in the US. They were noted with a lower occurrence risk of lung, bronchus, larynx, leukemia, neoplasms of the pharynx, oral cavity, esophagus, stomach, colon, rectum, liver, gall bladder, and pancreas, 48-52. For patients of PC, it is not entirely clear about the phenomenon of risk reduction, but we think that the old age at the time of diagnosis of PC may be one of the reasons 39. The mean age of PC diagnosis is about 67 years 53; the elder who has PC might not have the same chance of a second diagnosis as all US men 39. This might be an artifact of case-finding because advanced age at initial diagnosis of PC is associated with an underascertainment of second cancers 54. However, enhanced surveillance and screening after a PC diagnosis may have early detection of certain cancers, which causes a false phenomenon of the increased risk 39.

RT is used as a definitive treatment strategy in approximately 25% of localized PC patients 55. In general, exposure to ionizing radiation is considered to be a potential cause of cancer 2. Previous studies using one same SEER database have produced conflicting results concerning the risk of rectal cancer after RT for PC. Bexter *et al.* found a 1.7-fold increased risk of developing subsequent rectal cancer in patients treated with RT for PC compared to those treated with RP 3, while Kendal *et al*. could not demonstrate a similar outcome 56. Huo *et al*. expanded the SEER data until 2005 and demonstrated that there was indeed an increased risk for rectal cancer, but only after > 10 years of follow-up 57. Likewise, some studies reported an increased risk for rectal cancer after prostate irradiation 2, 25, 26, 39, whereas others found no association 22, 58-60. Possible reasons for this discrepancy are a limited sample size or short follow-up, differences in lag periods, and variation in statistical methods 61.

In principle, radiation-induced second cancers are defined as those cancers occurring inside or close to radiation-exposed regions (field congruence) 62. Generally, secondary cancers are predominantly expected to occur in the rectum and rectosigmoid colon than other parts of the colon. For evaluation of the radiation effect on the risk of developing secondary colorectal cancer, Baxter *et al.* compared the related data of three different sites: rectum, rectosigmoid/sigmoid/cecum, and the rest of the colon according to the risk of radiation exposure. They noted a significantly increased risk of rectal cancer among men who received RT, with a hazard ratio of 1.7 (95% CI, 1.4-2.2), in comparison to the male who did not, but there was no increased risk of cancer in the rest of the colon 3. However, Hegemann* et al*. showed that there was a 70% higher risk of rectal cancer in patients with RT compared to those treated with RP only and a less increased risk of colon cancer 4. Moon* et al*. even found that patients treated with RT had significantly higher odds of developing both rectum and sigmoid colon cancer (OR 5 1.60; and OR 5 1.26, respectively; *p* < 0.05) 19. However, all these results of SCRC were different from the conclusion of Mc Master *et al*. after re-evaluation of the SEER database, which showed no increased risk for either colon or rectum cancer. However, we had the same conclusion as that of Mc Master et al. Besides the possible causes mentioned above, the differences in the results among these studies may also be related to differences in the study design and analysis, outcome of interest, comparison population, measure of risk, etc. 59

Radiation-induced second cancers manifest with a frequency that could be obscured by the background incidence of spontaneous second cancers. This frequency seems so low as to make prospective randomized comparisons very difficult, and the potential for unrecognized confounders is such that the retrospective analysis of large population databases can be complicated 63. Thus, the effects of therapeutic radiation and the potential for radiation-induced second cancer should be considered in the context of the background of spontaneously occurring cancer, particularly in older populations. However, there is no specific marker available to allow a precise distinction between radiation-induced second cancers and non-radiation induced second cancer; all assessments are based on epidemiological and/or statistical analysis 4. Based on these reasons, many experts have suggested men undergoing radiation for PC should undergo regular endoscopic evaluation 3, 26.

Latency periods represent the time from radiation exposure until the diagnosis of subsequent cancer and latency period thresholds have been introduced to second cancer analyses to reduce possible bias from synchronous tumors. Unfortunately, there is no consensus regarding the length of time, after which malignancies are considered secondary to RT 4, 25. Between studies, the latency period thresholds in the reviewed studies vary from 1 month up to 15 years, often without evidence supporting the time thresholds 16, 17, 39, 58, 64, 65. This may have led to a discrepancy in the outcomes 25. Nonetheless, it is debatable whether the length of latency periods is the same, disregarding the patient's age or specific organs at risk 66, 67. Therefore, a fixed latency period is often introduced to reduce the chances of such bias 61. Some studies reported on multiple fixed latency periods and could, therefore, be entered into the meta-analysis repetitively. There was a significant increase of rectal cancers in time since primary treatment with calculated relative risks (RRs) for developing rectal cancer of 1.31 (95% CI 1.104-1.66), 1.51 (95% CI 0.97-2.33), 1.95 (95% CI 1.51-2.53), and 2.49 (95% CI 1.48-4.19), for 0-2 months, up to 5 years, up to 10 years and up to 20 years respectively (*p* = 0.0006) 61. Until this consensus regarding the latency period is clarified, it will remain debatable whether the subsequent rate of malignancies can be interpreted as clinically meaningful 25. In this study, we applied a shorter interval (3 years) to exclude the possibility of “synchronous” colorectal cancer. First, because the pathways leading to colorectal carcinogenesis in this unique setting remained unknown, any cutoff was arbitrary, and cancer occurring after three years was probably as much a secondary malignancy as a one occurring after five years. Secondly, from a patient perspective, any increase in morbidity after a specific treatment was essential to acknowledge whether genuinely secondary to the applied therapy or not.

In Table 2 of our study, we noted a definite risk of SCRC for the patients under RP compared with those among RT, especially cases in clinical stage I (HR: 0.14; 95% C.I.:0.01-1.39; *p* =0.0929). This phenomenon of borderline significantly protective effects of SCRC on patients with RP is hard to clarify. However, we think it may be related to the longer life expectancy of stage I PC patients than those of other stages, and no exposure to the radiation injury before. Thus they could obviate the risk of radiation toxicity effect on SCRC occurrence in a longer follow-up.

Our study represented a significant improvement over many prior institutional analyses of the SCRC after RT for PC. First, our study was based on a large, population-based design, and our results benefited from well-defined data collection and excellent quality standards designed to ensure that all eligible cases were selected, which allowed our results to be generalized across the Asian countries. Second, our population benefited from universal national health insurance. Therefore, diagnostic rates may differ from those populations in which economic and health care considerations may prevent the same rate of use and access to health resources, as was the case in the Western countries. Third, the uniqueness of our findings related to their origin. Most of our patients originated from a genetically, environmentally, and health economically similar population, compared with virtually all previous studies.

However, the current study was, in part, limited by the fact that the RT group included patients treated with all forms of radiation therapies, including conventional external beam RT, 3-dimensional conformal therapy, intensity-modulated radiation therapy, brachytherapy, and combination radiation therapy. Thus, the risk of subsequent colorectal cancer associated with these particular forms of radiation therapies could not be investigated explicitly by our retrospective studies, despite the fact that most patients had received conventional external beam RT during our study period. Second, data on the dosage or field of radiation are unavailable, and we were not aware of the doses of radiation administered to patients who received RT and, therefore, a dose-risk relationship of RT with SCRC could not be assessed. Third, we did not control for colorectal cancer risk factors such as a change in lifestyle after prostate cancer therapy, smoking habits, or family history. Fourth, we did not examine other adverse effects of RT and RP.

## Conclusion

Our study did not show differences in SCRC incidence between patients with PC treated with RP or RT after hazard adjustment by different stratum. However, this study should not be interpreted to discount the occurrence of radiation-induced tumors or provide data to change the guidelines for surveillance for colorectal cancer in previously irradiated patients.

## Figures and Tables

**Table 1:** Characteristics of prostate cancer (PC) patients

Variable	Prostate cancer with RP (N=5578)	Prostate cancer with RT (N=3219)	P-value
Age at diagnosed, years, n(%)			
<60	695(12.46)	190(5.90)	<.0001
60-69	1999(35.84)	763(23.70)	
≥70	2884(51.70)	2266(70.39)	
Clinical stage, n(%)			
I	889(15.94)	130(4.04)	<.0001
II	3257(58.39)	1639(50.92)	
III	662(11.87)	737(22.90)	
IV	770(13.80)	713(22.15)	
Comorbidity, n(%)			
Crohn's disease	3(0.05)	8(0.25)	0.0230
DM	1139(20.42)	655(20.35)	0.9360
ESRD	164(2.94)	116(3.60)	0.0877
Hyperlipidemia	1200(21.51)	683(21.22)	0.7450
Autoimmune	1046(18.75)	719(22.34)	<.0001
Gout	332(5.95)	202(6.28)	0.5408
Diffuse diseases of connective tissue	26(0.47)	14(0.43)	0.8341
Rheumatoid arthritis	31(0.56)	20(0.62)	0.6965
Osteoarthrosis and allied disorders	724(12.98)	525(16.31)	<.0001
LC	296(5.31)	183(5.68)	0.4512
COPD	633(11.35)	409(12.71)	0.0577
SCRC, n(%)	53(0.95)	32(0.99)	0.8392
Colon	44(0.84)	25(0.78)	0.5760
Rectum	9(0.16)	7(0.22)	0.5519
Time to SCRC after survived 2 years, median (Q1-Q3)	1.39(0.36-2.25)	0.91(0.40-1.90)	0.4661
Mortality, n(%)	996(17.86)	722(22.43)	<.0001
Time to death, after survived 2 years, median(Q1-Q3)	1.55(0.69-2.91)	1.72(0.80-3.00)	0.0627
Subjects with hormone therapy, n(%)	2270(40.70)	2463(76.51)	<.0001
Radiation colitis after PC, n(%)		122(3.79)	

**Table 2:** Adjusted hazard ratio of SCRC and rectal after therapy by different stratum

RP vs RT	Risk of SCRCHR (95% CI)*	P-value	Risk of rectal cancerHR (95% CI)*	P-value
All subjects	1.47(0.87-2.49)	0.1468	1.44(0.47-4.42)	0.5209
Age at diagnosed, years				
<60	0.83(0.02-38.88)	0.9221	-	
60-69	1.07(0.21-5.41)	0.9368	0.43(0.03-6.90)	0.5518
70≧	1.44(0.82-2.53)	0.2074	1.74(0.51-5.96)	0.3785
Clinical stage				
I	0.14(0.01-1.39)	0.0929	0.34(<.01-71.68)	0.6951
II	1.92(0.93-3.95)	0.0775	1.52(0.35-6.55)	0.5717
III	1.84(0.47-7.22)	0.3805	0.76(0.08-7.11)	0.8122
IV	0.64(0.19-2.08)	0.4535	-	
Comorbidity				
DM				
Yes	1.29(0.33-5.10)	0.7158	0.19(<.01-40.18)	0.5419
No	1.51(0.85-2.67)	0.1607	1.80(0.56-5.85)	0.3259
ESRD				
Yes	0.71(<.01-230.51)	0.9060	0.71(<.01-230.51)	0.9060
No	1.43(0.85-2.42)	0.1822	1.28(0.41-4.02)	0.6744
Hyperlipidemia				
Yes	1.32(0.25-6.89)	0.7427	0.43(<.01-80.22)	0.7505
No	1.49(0.85-2.59)	0.1624	1.65(0.51-5.37)	0.4039
Autoimmune				
Yes	2.98(1.00-8.88)	0.0500	1.59(0.08-31.48)	0.7609
No	1.15(0.63-2.11)	0.6445	1.32(0.39-4.53)	0.6561
LC				
Yes	5.84(0.38-90.17)	0.2064	1.64(0.01-510.44)	0.8652
No	1.34(0.78-2.29)	0.2919	1.28(0.41-4.00)	0.6778
COPD				
Yes	9.54(0.27-334.38)	0.2140	2.89(0.02-545.35)	0.6920
No	1.31(0.77-2.24)	0.3264	1.26(0.40-4.03)	0.6943
Hormone therapy				
Yes	1.28(0.69-2.39)	0.4339	1.55(0.44-5.47)	0.4993
No	1.59(0.54-4.68)	0.3972	0.62(0.06-6.63)	0.6930

*The HR was adjusted by age, clinical stage, and the listed comorbidities.
